# Risky alcohol use among reproductive-age men, not women, in Mae La refugee camp, Thailand, 2009

**DOI:** 10.1186/1752-1505-6-7

**Published:** 2012-09-11

**Authors:** Nadine Ezard, Supan Thiptharakun, François Nosten, Tim Rhodes, Rose McGready

**Affiliations:** 1 Department of Social and Environmental Health Research, Faculty of Public Health and Policy, London School of Hygiene and Tropical Medicine, 15-17 Tavistock Place, London, WC1H 9SH, UK; 2 Alcohol and Drug Service, Inner City Health Programme, St Vincent’s Hospital, 390 Victoria St, Darlinghurst, NSW 2010, Australia; 3 Shoklo Malaria Research Unit, 68/30 Baan Tung Road, P.O. Box 46, Mae Sot, Tak 63110, Thailand; 4 Mahidol–Oxford Clinical Research Unit, Faculty of Tropical Medicine, Mahidol University, 420/6 Ratchawithi Road Ratchathewi, Bangkok, 10400, Thailand; 5 Centre for Clinical Vaccinology and Tropical Medicine, Churchill Hospital, Oxford, OX3 7LJ, UK; 6 Centre for Research on Drugs and Health Behaviour, Faculty of Public Health and Policy, London School of Hygiene and Tropical Medicine, 15-17 Tavistock Place, London, WC1H 9SH, UK

**Keywords:** Alcohol, Substance abuse, Refugees, Assessment, Displaced population, Conflict, mSASQ

## Abstract

**Background:**

Globally, alcohol use contributes to close to 4% of all deaths and is a leading cause of ill health and premature death among men of reproductive age. Problem alcohol use is an unaddressed public health issue among populations displaced by conflict. Assessing the magnitude of the problem and identifying affected groups and risk behaviours is difficult in mobile and unstable populations.

**Methods:**

From 15–28 December 2009 we conducted a simple rapid screening test of risky alcohol use using the single item modified Short Assessment Screening Questionnaire (mSASQ) by all women currently enrolled in the antenatal care clinic in Mae La refugee camp, a long standing displaced setting on the Thai Burma border. Women self- reported and gave a secondary report of their male partners. Gender differences in alcohol use were further explored in semi-structured interviews with camp residents on attitudes, behaviours, and beliefs regarding alcohol and analysed thematically.

**Results:**

Of 636 women screened in the antenatal clinic, almost none (0.2%, 95CI 0.0-0.9%) reported risky alcohol use prior to pregnancy, whereas around a quarter (24.4%, 95CI 21.2-27.9%) reported risky alcohol use by their male partners. Interviews with 97 camp residents described strong social controls against women’s alcohol use and men’s drinking to intoxication, despite a dominant perception that the social context of life in displacement promoted alcohol use and that controls are loosening.

**Conclusions:**

As a stigmatised behaviour, alcohol use is difficult to assess, particularly in the context of highly mobile adult male populations: the simple assessment methods here show that it is feasible to obtain adequate data for the purposes of intervention design. The data suggest that risky drinking is common and normalised among men, but that the population may have been partially protected from rapid rises in problem alcohol use observed in nation-wide data from Thailand. The changing social context contains vulnerabilities that might promote problem alcohol use: further investigation, ongoing monitoring, and development of targeted interventions are warranted.

## Background

Globally, alcohol consumption is the third most important risk factor for disease and disability, responsible for close to 4% of all deaths (6% among men and 1% among women [1], although alcohol-related mortality is increasing among women [2]). Among males aged 15–59 years it is the leading cause of death; in middle income countries the World Health Organization reports alcohol use as ‘the greatest risk factor for disease and disability’ [1].

Little is known about alcohol use among displaced populations [3,4]. Data suggest that risky alcohol use is prevalent in some, but not all, war-displaced settings (such as Colombia [5] and Croatia [6]). One study showed that displaced high school students in Bosnia-Herzegovina were more likely to report alcohol use than non-displaced [7]. Alcohol use behaviours are context specific, related to a range of pre- and post-displacement influences (such as cumulative exposure to traumatic events [3,8]); limited access to services may exacerbate the harmful consequences. Wide-spread use of alcohol – particularly artisanal alcohol – is believed to be linked to a range of individual and community-harms by some long-term displaced populations in Kenya, Liberia, Uganda and Thailand [9].

The term ‘risky alcohol use’ is used here to mean alcohol consumption that increases the risk of alcohol-related harm (also called hazardous drinking), as well as alcohol consumption that is already causing alcohol-related harm (also called harmful drinking) [10]. Poor populations – particularly those living in southeast Asia – suffer more harm per gram of alcohol consumed than wealthy populations, due partly to poor access to health care and to harmful patterns of heavy episodic drinking [2]. Excess mortality due to alcohol occurs mainly in younger age groups (chiefly due to injury, and the lack of protective effects seen in older age groups) [2]. Similarly, most alcohol-attributable burden of disease occurs in early and middle adulthood: in other words, in people of reproductive age. There are a number of interventions with evidence of effectiveness elsewhere for low- and middle- income countries targeting risky (but not dependent) drinking [11]. Such an intervention approach marks a shift from provision of more complex interventions for alcohol dependence towards primary care- based early interventions, with greater potential population impact.

This paper reports on a rapid assessment of alcohol use conducted among refugees from Burma living in Mae La refugee camp, Thailand. Mae La camp is situated on Thailand’s western border with Burma. The border marks a difference in national economies: the World Bank classifies Thailand as a middle-income country, whereas Burma is classified as a low-income country [12]. Intrastate conflict in Burma has displaced more than two million people into neighbouring Thailand since independence from the UK in 1948 [13]. Militarised ethnic conflict has resulted in the forced displacement of minority ethnic groups, such as the Karen, by the dominant Burman ethnic group [14]. At the time of the study, more than 160,000 refugees resided in nine camps along the border [15,16], in addition to around 500,000 displaced internally in Burma [17] and several million undocumented and documented migrant workers from Burma in Thailand. Mae La is the largest of the refugee camps, established in 1984 and home to approximately 45,000 people [18]. The majority of the population is Karen-speaking and of Buddhist and Christian religions, while Burmese-speaking Muslim Karen make up around 14% [19]. Employment and education opportunities are limited, and the population is largely dependent on external aid (supplemented by casual labour, stipends, and a growing importance of third-country remittances) [19]. There is high population turnover. A program of third country resettlement began in 2005, with new arrivals replacing the approximately 20,000 who had departed (as of Jan 2010 [20]). Access to primary health care and education is supported by non-government organisations; in addition there is an abstinence-based residential substance use treatment programme in the camp. Health indicators for the camp are intermediate between the Thai population and estimates from eastern Burma [16,21,22].

Although no prevalence data on use and harm are available for Mae La, alcohol is an important public health and social concern for the population. Alcohol is readily available and its use tolerated, particularly the cheap artisanal rice liquor, despite its illicit status in Thailand. Beer, wine and whisky can be obtained at nearby bars and shops, although not officially permitted to be sold in the camp [9]. Population surveys show that alcohol use is seen as an important security concern in Mae La [23-25]. Service provider data listed alcohol as an important cofactor in recorded incidents of gender-based violence, physical assault and suicide [26].

There are a number of methodological constraints to conducting a ‘gold standard’ household prevalence survey [27] to describe the distribution of alcohol use patterns and harm and to identify the most affected populations in displacement settings. Previous agency reports from Mae La suggested considerable stigma against substance use and fear of retribution [28]: likely under-reporting due to fear of stigmatization has been noted in other conflict affected settings (eg [29]). Furthermore, Mae La has a high population mobility [19] which may affect population size estimates and survey coverage. Young adult men – likely to be a most affected group – are particularly mobile [30]. Finally, the costs (and opportunity costs) involved in a household survey are not inconsiderable.

## Methods

### Systematic screening for risky alcohol use through the ANC clinic

We administered an adapted version of the modified Single Alcohol Screening Questionnaire (mSASQ [31], the third question of ‘gold standard’ 10-item Alcohol Use Disorders Identification Test (AUDIT [10]) in Burmese, Poe or Sgaw Karen to all pregnant women seen in the camp ANC clinic between 15 and 28 December, 2009. Since both volume and frequency of alcohol consumption affect health [2], we used a single screening question designed to capture frequency of risky high-volume drinking [31]. The time period was designed to capture all pregnant women enrolled in the programme, who are expected to attend at least every two weeks. The clinic did not close for Karen New Year (16 December) or Christmas Day (25 December) which fell during the study period.

To be consistent with the AUDIT, the same cut-off of six standard drinks (of approximately 10 g ethanol each) was used for women and men. The English version of the two-part question was:

"‘Starting from last year “Sweet December” [1 December – an important date for many Karen families] until before you got pregnant, how often did you drink six standard drinks or more than six standard drinks in one occasion?’ and"

"‘Starting from last year “Sweet December” until today, how often does your husband drink six standard drinks or more than six standard drinks in one occasion?’"

The answer monthly, weekly or daily or nearly daily was considered positive [32]. A photograph showing the number of standard drinks in beverages commonly consumed in Mae La (including usual measures of artisanal rice liquor and carbonated wine beverage marketed to women in Thailand) was used to aid in eliciting responses [see Additional file 1. We recruited, trained and supervised two women mental health workers who spoke Burmese and Karen to administer the questionnaire in the antenatal clinic. Anonymous responses were recorded onto record sheets for later data entry, and inclusion in the survey was marked on the clinic record.

### Individual key informant interviews

Key informants were defined as people living in Mae La with first-hand experience of alcohol use and other household members (most often the women partners of male alcohol users, as well as children, parents, and siblings affected by their use) [33,34]. Interviews were conducted by pairs of field workers in Burmese or Karen from a team of seven trained and supervised camp residents using a semi-structured interview guide (based on a literature review and experience elsewhere [35]), audio recorded, transcribed and translated into English. Participant data and observations were recorded during and immediately after the interview. Initial interviews lasted 60 minutes or less. Ten participants were invited for subsequent open-ended interview (range 20–80 minutes).

Sampling was purposive, aiming to ensure diverse range of cultural experience to achieve cultural not demographic representativeness [36]. Based on available literature and conversations with the field team, we sampled to ensure a diverse range of participants by gender, religion, ethnicity, duration of residence in Mae La (‘new arrivals’ who arrived after resettlement commenced, and ‘long-term residents’ who arrived prior to resettlement and who are largely eligible for resettlement), and personal experience of alcohol use. Participants were recruited through health and substance use services, interviewer networks, and chain referral (asking participants to refer family members or friends, also known as snowball sampling). Recruitment of new participants stopped once the team believed that ‘data saturation’ had been achieved [37]; specifically, no substantively new information relating to alcohol use and no new obvious themes emerged over a three day period of interviewing (13 participants) using the semi-structured interview guide.

### Ethical considerations

Written approval was obtained from the Ethics Committee of the London School of Hygiene and Tropical Medicine prior to the study. All participants provided written informed consent. The importance of maintaining confidentiality was emphasised with all team members and translators, and they were instructed to ensure that no names appeared on any document; pseudonyms are used in this report. Participants were thanked with a small gift (soap, candles, toothbrush, or a non-alcoholic beverage) and invited to attend a community feedback meeting of the preliminary study findings.

### Analysis

Quantitative data were coded and entered into Epi Info^TM^ 3.5.1 (Centers for Disease Control and Prevention, Atlanta, Georgia, USA) and analysed using STATA® v11.1 (Statacorp, College Station, Texas, USA) for simple frequency count and proportions (with 95% confidence intervals calculated using the Wilson score method [38,39]). Means of normally distributed data were compared using Student’s t test and proportions of categorical data using Pearson chi-square tests. Tests of association were considered significant at the p < 0.05 level.

Qualitative data were analysed thematically [40], using an iterative process, beginning simultaneously with data collection. Initial analysis was conducted by the team during the field work period. Systematic open coding of the full data set was made using Excel® software (Microsoft corporation, Redmond, Washington, USA), extracting major common themes or important divergent themes, and validated and refined by the team. Further analysis − including recoding, looking for linkages and connections between the first level codes, searching for negative or deviant instances and examining material by speaker attribute − was completed with the aid of the qualitative software NVivo8® (QSR International, Doncaster, Victoria, Australia). Quotations were extracted to illustrate key themes.

## Results

### Antenatal clinic survey

There were 636 participants, representing a coverage of 89.8% of those enrolled in the ANC clinic for that period (midpoint of weekly registrations for the two week study period, 708, which represents a coverage of approximately 8.1% (708/8729) of the population of women of childbearing age, 15 to 49 years). The majority of interviews were conducted in Sgaw Karen (58.8%; 374/636), the remainder in Burmese (25.8%; 164/636) or Poe Karen 15.4% (98/636). Women reported their ages as 15 to 47 years (median age 26.2 years); their male partners as 17 to 65 years (median age 29.0 years). Data were not collected on other variables such as duration of residence in Mae La.

A significantly higher proportion of women screened their male partner’s drinking as positive for risky (binge) drinking than their own drinking (p < 0.001). The proportion of women screening positive was negligible (0.2%, 1/636, 95CI 0.0-0.9%); the proportion of women screening their male partners positive was much higher at around a quarter (24.4%, 155/636, 95CI 21.2-27.9%). Break down by score and by gender are given in Table 1. The proportion of women who screened their male partners positive was lower among Burmese speakers (13.4%) than Poe Karen (26.5%) or Sgaw Karen (28.6%) speakers (p < 0.001). There was no difference in mean age of the three language groups (p = 0.56). Males who scored positive were significantly more likely to be 25 years of age or older, than younger than 25 years; the highest prevalence was observed in the 40–44 year age group (Table 2).

**Table 1:** Frequency of consumption of 6 drinks or more by gender, Mae La ANC, 2009

	**Women (self-report)**				**Men (secondary report)**			
**Score**	**Frequency**	**Percent**	** 95CI**		**Frequency**	**Percent**	** 95CI**	
Never	624	98.1%	96.7%	98.9%	358	56.3%	52.4%	60.1%
Less than monthly	11	1.7%	1.0%	3.1%	123	19.3%	16. 5%	22.6%
Monthly*	1	0.2%	0.0%	0.9%	79	12.4%	10.1%	15.2%
Weekly *	0	0.0%	-	-	41	6.4%	4.8%	8.6%
Daily*	0	0.0%	-	-	35	5.5%	4.0%	7.6%
Total	636	100%	-	-	636	100%	-	-

Consumption defined as at least 6 standard drinks of approximately 10 g ethanol each in one session over the previous 12 months for women (on self-report, pre-pregnancy) and men (on secondary report).

*indicates positivity for risky drinking.

**Table 2:** Positive screening result for risky drinking using the mSASQ by age group and gender, Mae La ANC, 2009

**Age group**	**Women (self-report)**				**Men (secondary report)**			
**(years)**	**n**	**Positive**	**95CI**			**Positive**	**95CI**	
**15-19**	113	-	-	-	26	11.5%	4.0%	29.0%
**20-24**	165	-	-	-	141	19.1%	13.5%	26.4%
**25-29**	165	-	-	-	170	25.3%	19.4%	32.3%
**30-34**	111	-	-	-	142	23.2%	17.1%	30.8%
**35-39**	62	1.6%	0.3%	8.6%	87	28.7%	20.3%	39.0%
**40-44**	17	-	-	-	44	38.6%	25.7%	53.4%
**45+**	3	-	-	-	26	26.9%	13.7%	46.1%
**Total**	636	0.2%	0%	0.9%	636	24.4%	21.2%	27.9%

mSASQ: modified Single Alcohol Screening Questionnaire.

### Interview data

Interviews with 97 key informants were conducted from 11 September to 21 December 2009 (Table 3).

**Table 3:** **Interview sample, Mae La refugee camp, 2009** (n = 97)

**Characteristic**		**n (%)**
**Gender**	Women	32 (32%)
**Alcohol use**	Current alcohol users	75 (77%)
	-of which women	9 (12%)
**Religion**	Buddhist	19 (20%)
	Christian	69 (71%)
	Muslim	4 (4%)
**Ethnicity**	Sgaw Karen	72 (74%)
	Poe Karen	12 (12%)
	Muslim Karen	1 (1%)
	Burman	4 (4%)
**Age group**	15-20 years	13 (13%)
	20-44 years	58 (60%)
	≥ 45 years	26 (27%)
**Duration of residence**	0-4 years	55 (57%)
	≥5 years	42 (43%)

Artisanal rice based distilled alcohol was the predominant alcoholic beverage in the camp, (costing about Baht 5–10, or 10–20p in UK currency at October 2009 rates, for the equivalent of approximately 10 g ethanol^a^). Despite its illicit nature, artisanal alcohol was easily available, although hidden. There was a dominant belief that the pressures of displacement and refugee camp life promoted alcohol use. Mae La residents ‘have only alcohol. If they go out, the Thai will catch them. Here, it’s like being in a farm. It is surrounded by a fence, they can’t go out, so if they get upset, there’s only alcohol to get release’ Saw Y explained (a 24-year-old man who had been resident for two years). He went on to list the constrained options for many people. ‘Some people stay here and they feel trapped. If they go back to Burma, they will be arrested. Living like that, they don’t know what to hope for. So they just drink alcohol.’ Many participants suggested that a pervasive sense of hopelessness drove alcohol use. Saw V for example linked this feeling directly to his drinking ‘I have no goal. I don’t know the future. At that time, I drank only alcohol … When does the UN call me? By waiting like this, I lost my goal. So I drank alcohol. After that, time flies by a day at a time’. On the other hand, a sense of hopefulness provided by access to education and resettlement were seen as preventing problem alcohol use.

Yet these pressures were rationalised as driving men’s, not women’s, drinking, in a context where drinking behaviours and norms were gendered. For example, our sample included seven women who drank regularly (at least once a week) who considered themselves and were considered by others as a minority. Abstinence was associated with femininity; women were expected to exert more self-control than men. Naw P, a 22-year-old woman who had been in Mae La since the age of 17, explained that: ‘[men drink] because they get tired, they keep drinking until they are drunk in the street. Women also get tired! Drinking alcohol to freshen up, that is just giving into your desires’. Saw Y, a 24-year-old man, thought that there were fewer women than men who drink because: ‘women can control themselves if they get upset. Usually, men have no self-control’. For some, this ‘self- control’ was driven by strong normative pressures against women’s alcohol use, and a fear of social exclusion for contravening social norms. As Saw V, a 20-year-old man, explained: ‘they are afraid that the neighbours will gossip about them’. Sometimes religious norms were invoked in explanation for proscription of women’s drinking. For example, Maung T, a 22-year-old man and himself a regular drinker, explained that women should not drink ‘because they are females. It is not suitable for our [Muslim] religion, it is not accepted. It’s not good to see.’

Alcohol use by men was subject to a number of social controls that predate displacement. Karen people have long produced alcohol. Saw P, a 56-year-old man who had been living in and out of the camp since it was established, explained: ‘alcohol drinking is not unusual for the Karen people, the Karen people drink alcohol based on their custom such as weddings, funerals and so on … they drank alcohol in these situations but had no problems with alcohol drinking’. Alcohol use was a social activity, and it was often referred to in Karen as ‘happy water’. According to Saw S, this means ‘it is like we drink alcohol in order to make us happy but I do not know how to explain it. Maybe some people might have a different meaning of it. As for me I have a lot of friends sometimes we buy a bottle of alcohol and drink together with friends and come back to eat after drinking but I do not continue to have more. I just drink it in moderation.’ In addition to aiding in socialisation and mood, there was a dominant perception that small amounts of alcohol use were beneficial for physical health. We heard repeatedly that ‘alcohol is good to eat rice’ (improves appetite) and ‘alcohol is like medicine’ (improves health). The concept of ‘drinking in moderation’ appeared to be core to culturally acceptable use, while intoxication and dependence were stigmatised. As Saw E, a 43-year-old man and long term resident, explained: ‘[if alcohol is drunk] within limits, it is like medicine. If it is over the limit, it is dangerous.’ Another example of drinking over the limit was being loose-tongued and indiscreet described frequently as: ‘the theft of the buffalo is revealed’

Occasional alcohol use was described as a culturally appropriate aid to socialisation, and perhaps even a usual part of transition to adulthood, for men. Intoxication and dependence, on the other hand, were proscribed, as was drinking by women. However, these social controls were described as changing under the pressures of displacement and refugee camp life. In particular, young people and women were now thought to drink more often and more prominently, and alcohol was used more often and more often to intoxication or dependence. Saw A, an 80-year-old man who had been resident for 17 years, expressed the dominant perception: ‘Now more and more people are drinking alcohol’. Various explanations were given, including exposure to new cultures and alcohol use behaviours, on-going population movements, increased social diversity in the camp, increased economic resources derived from third country remittances, and changing social networks. Saw P continued: ‘before there were not so many people in the camp and there are fewer cases [of alcohol and drug problems] happening in our community … but now more and more people are coming into the camp with different ethnic backgrounds and different characteristics and more fighting, more drugs, and more problems happen in the camp’.

## Discussion

The interview data suggest that the alcohol use culture in Mae La – like that from long-term displaced camps in Kenya and Uganda [9] – can be characterised as predominantly ‘mood changing’, as opposed to regular or ‘nutritional’ low volume drinking with meals [41]. Mäkelä and colleagues’ use this term to describe cultures with a high prevalence of abstention and social unacceptability of alcohol use, but where drinking does occur it may be to intoxication [42]. As in Mae La, drinking in such cultures is associated with masculinity, and abstinence with femininity and submission [43].

The clinic survey data confirm marked gender differences in reported alcohol use. Since the majority of adult women and men living in Mae La are engaged in human reproduction and antenatal coverage is high, the findings from the antenatal clinic study have some implication for population prevalence among this age group. Results showed that almost no women reported binge drinking prior to pregnancy whereas around a quarter reported at least monthly binge drinking episodes by their male partners. The interview data suggests that alcohol use by women was seen as aberrant, and the shame and stigma associated with women’s public drinking, drunkenness and dependence was much greater than men’s.

Alcohol use shows similar differences by gender in Thailand. Although not directly comparable, recent national survey data from 2007 using a different screening instrument (the full AUDIT) suggest that 23.8% of men and 1.5% of women in the 25- to 44-year-old age group the Thai population drink alcohol at risky levels [44]. No data are available for the country of origin, Burma. Data from the UK – a resettlement country – using a slightly different version of the mSASQ (including cut-offs for men and women 48 g and 64 g ethanol respectively) suggest a prevalence of around a third in primary care settings, higher than the prevalence we observed in Mae La [45].

Highlighting the complexity and context-specificity of alcohol and displacement, risky alcohol use among this population may be lower than other displaced populations in alcohol-drinking cultures. Alcohol dependence (using a different screening tool than the AUDIT) was reported to be as high as 61% of men and 8% of women in one displaced population in Croatia [6]. Data from long-term camps for the internally displaced in northern Uganda using the full AUDIT suggest that 32% of men and 7% of women drink alcohol at risky levels [8] – the authors tentatively conclude here also that the use of alcohol among displaced populations is not higher, and may be slightly lower, than that of the general population in Uganda.

Similar gender differences were shown in systematic screening of inpatients in Mae La conducted during the same period. Around a third of men (30.8%, 12/39, 95CI 18.6-46.4%) and no women (0/38; 95CI 0.0-9.2%) screened positive using the full AUDIT [46]. These findings were similar to a Thai emergency room study conducted more than 10 years earlier, in 1998, using similar methods. In this study 39% of the men and 8% of the women participants screened positive using the AUDIT (n = 933) [47]. It is likely that prevalence in Thailand would be higher in 2009: Thailand has experienced a marked increase in problem alcohol use in over the past 10 years, in the context of increased GDP and vigorous alcohol marketing [48]. Uptake is particularly rapid among young women [49]; while risky alcohol use remains more prevalent among men [44].

The interview data suggest a number of features of life in displacement that might promote high risk drinking in Mae La, such as a sense of powerlessness and hopelessness. Various models have been applied to explain how these features might be expressed in substance use. Examples include the social stress model [50,51] whereby social stressors may promote individual substance use proposed by Galea and colleagues [52]. Alternatively, a ‘self-medication’ hypothesis [53] has been proposed by Singer [54], whereby drugs are used to relieve individual suffering.

Yet these models do not explain why the observed prevalence of high risk drinking was not higher. Such pressures might be countered by the strong social controls that were reported to limit high risk drinking: in addition to proscription of drinking by women, intoxication, drinking by young people and solitary drinking are also proscribed. In the past, qualitative data have suggested that the relative geographical and social isolation of the camps has been partially protective against unsafe behaviours such as sexual behaviours risky for sexually transmission infection, but this situation is changing with the growth of the camp, increased contact with populations outside the camp, and more people moving in and out of the camp to look for work [55]. The qualitative data presented here also suggest that there is a perception that there is some transition of alcohol use behaviours from low-risk ‘traditional’ to high-risk consumer oriented. Efforts to prevent transition to more harmful use can be guided by more investigation into why the prevalence of high risk alcohol use in the camp is not higher, in line with growing research on community resilience [56]. For example, tight social networks have been suggested to be partially protective from problem substance use in some conflict-affected populations [57].

There were several important limitations to the study. The cut-off of six standard drinks of approximately 10 g ethanol per drink for both men and women was used as it is coherent with the usual version of the AUDIT that has been validated in many settings internationally [10]. In this setting where most alcohol is artisinally-produced and home-poured, any assessment of standard drinks is at best an approximation.

The use of a single item screening test offered number of advantages: it is non-invasive, quick and simple to administer in a resource poor setting. This is the first time, to our knowledge that the mSASQ has been adapted to this setting. Nevertheless, bias may have resulted from the process of adaptation and translation, and data on validity and reliability of this measure in this population is not known; more work is required. Furthermore, the mSASQ is a screening test for a health risk, not a consequence. Screening tests are designed to be sensitive (aiming to detect all of those who drink alcohol at risky levels) at the expense of specificity (accepting that many people will be screened as positive who do not drink alcohol at risky levels), biasing the results towards over-estimation of the prevalence of risky drinking.

The data show women’s *perceptions* of their male partners’ use. Research from other settings suggests that family members may underestimate the amount that others are drinking [58]. Nevertheless, women have been asked about their male partners’ drinking elsewhere with good correlation of the AUDIT [59]. Women’s secondary report is a useful way to access data on hard-to-access populations such as mobile young men. Data represent women’s *report* of their own and partner’s use; concern about possible negative consequences may act as a disincentive to disclosure of high levels of use. Survey response accuracy is known to be influenced by the social context of questionnaire administration [60]. While all surveys are subject to these kinds of biases, they may operate particularly strongly in the refugee camp setting and where confidence in the confidentiality of the research process is perhaps low. These factors may bias the results towards an under-estimation of the prevalence of risky alcohol use.

Caution should be made when attempting to generalise the findings to the whole population of reproductive-age in Mae La, or other camps on the border. The study population was pregnant women attending the clinic and their male partners. Pregnant women who do not attend the clinic, women who are not pregnant and men who are not partnered with pregnant women are excluded: it is not known if excluded populations use alcohol in different ways than the included population.

The illicit nature of alcohol and its place in the social and economic life of the camp made it a sensitive topic to explore, compounded by difficulties in finding adequate private space in which to conduct the interviews. Other more in-depth ethnographic techniques are required [61] to explore the role that alcohol use plays in creating and defining gender [61]. More social science research on social networks and drinking contexts is indicated, as is economic analysis of alcohol use: in low and middle income alcohol-using populations, purchasing power is strongly related to alcohol use [1].

## Conclusions

Accessing the young adult population through the antenatal clinic represents a very quick, simple, low resource approach to obtaining some quantitative data in the absence of more comprehensive data on alcohol use. The findings suggest that risky alcohol use among reproductive-age men (but not women) is common and warrants targeted early intervention. The data do not suggest that risky drinking prevalence is greater than that of the Thai population. Rapidly obtained qualitative data suggest that cultural controls against women’s drinking and against men drinking to intoxication that predate displacement remain strong despite the chronic nature of displacement. Interventions aimed at strengthening these controls – which may partly explain why risky alcohol use is not more common even in the face of important social stressors – may be warranted, along with individual interventions such as sensitising curative health services to alcohol-related health problems and screening and brief intervention for risky but not dependent drinking, targeting young adult men [62,63]. While not replacing in-depth epidemiological or qualitative research, the results do suggest that detailed and complex social and medical research programmes are not necessary to begin to build experience in intervention in this important but neglected area of public health of risky alcohol use among conflict-displaced populations.

## Endnote

^a^Cost estimates calculated with an ethanol concentration of around 25% and usual packet size of around 40 ml. Estimates of alcohol concentration were based on measurements taken from four samples of the commonly used artisanal distilled rice liquor (23%, 26%, 27%, 29% ethanol, respectively, 0% methanol; SGS (Thailand) Laboratory Services, gas chromatography/flame ionization detector, 14 September 2009.)

## Abbreviations

AMI: Aide Médicale Internationale; ANC: Ante-Natal Care; AUDIT: Alcohol Use Disorders Identification Test; KWO: Karen Women’s Organisation; mSASQ: Modified Single Alcohol Screening Questionnaire; SMRU: Shoklo Malaria Research Unit.

## Competing interests

The authors declare that they have no competing interests.

## Authors’ contributions

RM conceived of the ANC study, participated in its design, coordination and analysis and helped to draft the manuscript. NE conceived the overall study, and was responsible for its design, implementation, management, analysis, and write-up. TR participated in the ANC study coordination, tools development, and data collection. FN and TR participated in the study conception and manuscript preparation. All authors read and approved the final manuscript.

## Supplementary Material

Additional file 1 Standard drink estimation pictoral aid, Mae La, Thailand, 2009 photographs of some commonly used alcoholic beverages and the number of standard drinks contained. Click here for file
